# Testing of Auxotrophic Selection Markers for Use in the Moss *Physcomitrella* Provides New Insights into the Mechanisms of Targeted Recombination

**DOI:** 10.3389/fpls.2017.01850

**Published:** 2017-11-03

**Authors:** Mikael Ulfstedt, Guo-Zhen Hu, Monika Johansson, Hans Ronne

**Affiliations:** ^1^Department of Forest Mycology and Plant Pathology, Swedish University of Agricultural Sciences, Uppsala, Sweden; ^2^Department of Molecular Sciences, Swedish University of Agricultural Sciences, Uppsala, Sweden

**Keywords:** auxotrophic selection markers, complementation, imidazoleglycerol-phosphate dehydratase, phosphoribosylanthranilate isomerase, *Physcomitrella patens*, plasmid rescue, shuttle plasmid

## Abstract

The moss *Physcomitrella patens* is unique among plants in that homologous recombination can be used to knock out genes, just like in yeast. Furthermore, transformed plasmids can be rescued from *Physcomitrella* back into *Escherichia coli*, similar to yeast. In the present study, we have tested if a third important tool from yeast molecular genetics, auxotrophic selection markers, can be used in *Physcomitrella*. Two auxotrophic moss strains were made by knocking out the *PpHIS3* gene encoding imidazoleglycerol-phosphate dehydratase, and the *PpTRP1* gene encoding phosphoribosylanthranilate isomerase, disrupting the biosynthesis of histidine and tryptophan, respectively. The resulting *PpHIS3*Δ and *PpTRP1*Δ knockout strains were unable to grow on medium lacking histidine or tryptophan. The *PpHIS3*Δ strain was used to test selection of transformants by complementation of an auxotrophic marker. We found that the *PpHIS3*Δ strain could be complemented by transformation with a plasmid expressing the *PpHIS3* gene from the *CaMV 35S* promoter, allowing the strain to grow on medium lacking histidine. Both linearized plasmids and circular supercoiled plasmids could complement the auxotrophic marker, and plasmids from both types of transformants could be rescued back into *E. coli.* Plasmids rescued from circular transformants were identical to the original plasmid, whereas plasmids rescued from linearized transformants had deletions generated by recombination between micro-homologies in the plasmids. Our results show that cloning by complementation of an auxotrophic marker works in *Physcomitrella*, which opens the door for using auxotrophic selection markers in moss molecular genetics. This will facilitate the adaptation of shuttle plasmid dependent methods from yeast molecular genetics for use in *Physcomitrella*.

## Introduction

Much of our knowledge about basic functions of the eukaryotic cell such as the cell cycle, intracellular transport, and gene expression was obtained by research in the budding yeast *Saccharomyces cerevisiae*. The success of yeast as a model organism can be attributed to the sophisticated tools and methods that have long been available in yeast. First and foremost comes gene targeting by homologous recombination, a method that was invented in yeast, thus making reverse genetics possible (Orr-Weaver et al., 1981). Gene targeting also works in some multicellular eukaryotes, but with a much reduced efficiency as compared to yeast. The moss *Physcomitrella patens* is the only multicellular organism that has gene targeting whose efficiency is comparable to that in yeast (Schaefer and Zrÿd, 1997), a finding that helped to establish *Physcomitrella patens* as a model organism (Schaefer, 2002; Cove, 2005; Lang et al., 2008; Rensing et al., 2008).

A second important method in yeast molecular genetics is the use of shuttle plasmids that are able to replicate both in yeast and in bacteria (Beggs, 1978; Stinchcomb et al., 1979). Shuttle plasmids make it possible to clone genes from plasmid libraries by complementation of yeast mutants, and also enable more advanced methods such as dosage suppressor screens (Rine, 1991). Gene targeting and dosage suppression complement each other in that gene targeting looks at what happens when a gene function is lost, whereas dosage suppressor screens look at what happens when a gene is overexpressed. By using both methods much can therefore be elucidated about the function of a given gene. The dosage suppressor screen is a particularly useful tool, since new suppressor genes are cloned in the screen and are thus directly available for further studies, and since a suppressor screen can be carried out in a relatively short time. Theoretically it is possible to identify all proteins in a given signaling or metabolic pathway in one single dosage suppressor screen.

Several attempts have been made to develop shuttle plasmids for use in plants and animals, but they have been frustrated by the fact that plasmids transformed into plant or animal cells tend to rearrange and randomly integrate into the host chromosomes, making it difficult to recover (rescue) the original plasmid from transformed cells (Van Craenenbroeck et al., 2000). However, transformed DNA can replicate episomally in *Physcomitrella* (Schaefer et al., 1991; Knight, 1994; Schaefer, 1994; Ashton et al., 2000) and we showed in a previous study that plasmids transformed into *Physcomitrella* can be rescued back into *Escherichia coli* without having undergone deletions or rearrangements (Murén et al., 2009). This opens up the possibility of using shuttle plasmid-based methods in *Physcomitrella*.

A third important tool in yeast molecular genetics is the use of multiply auxotrophic yeast strains. These are strains that carry mutations in biosynthetic genes, typically genes needed for amino acid or nucleotide biosynthesis. Auxotrophic strains are therefore unable to grow unless the medium is supplemented with the necessary amino acid or nucleotide. With such strains, it is possible to select tranformants by using plasmids that carry a wild type copy of the mutated gene, provided that the reversion frequency of the mutation is lower than the transformation frequency. The latter condition is ensured by using non-reversible knockout mutations. The advantages of using auxotrophic selection markers is that they do not require the use of antibiotics that may have undesired side effects on the organism under study, and that there are many more auxotrophic markers than drug resistance markers, which facilitates experiments with several plasmids and/or gene knockouts in the same strain.

Previous work in *Phsycomitrella* (Ashton and Cove, 1977) and *Arabidopsis* (Last and Fink, 1988; Last et al., 1991; Muralla et al., 2007) has shown that it is possible to find auxotrophic mutant plants that require a given nutrient in classical genetic screens. In the present study, we use gene targeting to create stable auxotrophic knockout mutants in *Physcomitrella* that are suitable for reverse genetics and work with shuttle plasmids. This was done by knocking out the moss ortholog of the yeast phosphoribosylanthranilate isomerase gene, *ScTRP1*, needed for biosynthesis of tryptophan, and the moss ortholog of the yeast imidazoleglycerol-phosphate dehydratase, *ScHIS3*, needed for biosynthesis of histidine. As a proof of principle, we further show that the *PpHIS3*Δ knockout strain can be complemented by a plasmid carrying the wild type *PpHIS3* gene expressed from the *CaMV35S* promoter, and that such transformants can be selected directly on histidine-less medium. Finally we show that the original plasmid can be rescued from moss transformants back into *E. coli*. This shows that cloning by complementation is possible in *Physcomitrella* and that auxotrophic selection markers can be used for molecular genetics work in moss.

## Materials and Methods

### Plant Material and Growth Conditions

*Physcomitrella patens* of the Gransden Wood ecotype (Ashton and Cove, 1977) was grown on 0.8% agar plates containing BCD medium (Nishiyama et al., 2000), i.e., 1 mM MgSO_4_, 1.85 mM KH_2_PO_4_, 10 mM KNO_3_, 45 μM FeSO_4_, 1 mM CaCl_2_, and trace elements, supplemented with 5 mM ammonium tartrate. The *PpHIS3*Δ knockout strain was grown on the same medium but with 250–500 μM of added histidine and the *PpTRP1*Δ knockout strain was grown on medium with 75–200 μM of added tryptophan. Moss protonemal tissue was grown on plates covered with cellophane. The growth conditions were 25°C in a Sanyo MLR-350 light chamber under constant light at about 30 μmol m^-2^.

### Construction of the *PpHIS3*Δ and *PpTRP1*Δ Knockouts

To clone the flanking sequences of *PpTRP1*, primers MU59 (containing an *Hin*dIII site) and MU47 TRP1-5R (containing an *Xho*I site) (Supplementary Table S1) were used to amplify a 901 bp fragment that was cloned into the TA-cloning vector pCR^®^2.1-TOPO, thus producing pTRP1-5′. Primers MU60 (containing a *Not*I site) and MU49 TRP1-3R (containing a *Bgl*II and a *Hin*dIII site) (Supplementary Table S1) were used to amplify a 548 bp fragment that was cloned into the TA-cloning vector pCR^®^2.1-TOPO, thus producing pTRP1-3′. To clone the flanking sequences of *PpHIS3*, primers MU64 situated upstream of a native *Hin*dIII site and MU65 (containing an *Xho*I site) (Supplementary Table S1) were used to amplify a 1424 bp fragment that was cloned into the TA-cloning vector pCR^®^2.1-TOPO, thus producing pHIS3-5′. Primers MU63 (containing a *Not*I site) and MU57 HIS3-3R (containing a *Bgl*II site) (Supplementary Table S1) were used to amplify an 893 bp fragment that was cloned into pCR^®^2.1-TOPO, thus producing pHIS3-3′.

The plasmids pHIS3-5′ and pTRP1-5′ were cut with *Hin*dIII and *Xho*I. The fragments containing the *Physcomitrella* 5′ upstream sequence of *PpTRP1* and *PpHIS3* were then cloned into pBHRF (Thelander et al., 2007; Schaefer et al., 2010) using the *Hin*dIII and *Xho*I sites in the vector, producing plasmids pBHRF-HIS3-5′ and pBHRF-TRP1-5′, respectively. Plasmids pHIS3-3′ and pTRP1-3′ were cut with *Not*I and *Bgl*II. The *Physcomitrella* 3′ downstream sequence of *PpHIS3* from pHIS3-3′ was then cloned into pBHRF-HIS3-5′ and the *Physcomitrella* 3′ downstream sequence of *PpTRP1* from pTRP1-3′ was cloned into pBHRF-TRP1-5′ using the *Not*I and *Bgl*II sites in these plasmids, thus producing pBHRF-HIS3-5′-3′ and pBHRF-TRP1-5′-3′, respectively.

For the gene targeting, pBHRF-HIS3-5′-3′ was cut with *Hin*dIII and *Spe*I and pBHRF-TRP1-5′-3′ was cut with *Hin*dIII. The linearized plasmids were transformed into moss protoplasts using the polyethylene glycol method (Schaefer et al., 1991), but with 500 μM of histidine or 100 μM of tryptophan added to all solutions and plates. pBHRF-HIS3-5′-3′ transformants were selected in the presence of 30 μg/L hygromycin and 500 μM histidine. pBHRF-TRP1-5′-3′ transformants were selected on plates containing 5–30 μg/L hygromycin and 25–100 μM tryptophan. The concentrations of the supplemented amino acids proved to be important. Initially a concentration of 500 μM was used for both tryptophan and histidine. This was, however, growth inhibitory, particularly in the case of tryptophan, which also showed a negative interaction with hygromycin. We therefore lowered both the concentration of tryptophan and hygromycin in the selection plates. The resulting stable transformants were then screened for inability to grow on histidine-less or tryptophan-less media.

### Verification of Gene Disruptions

Disruptions were verified by PCR using the Advantage 2 PCR kit from Clontech (Mountain View, CA, United States). For *PpHIS3*Δ, primers MU70 HIS3-5F and 35S-R (Supplementary Table S1) were used to verify 5′-end integration of the knockout construct, and primers MU71 HIS3-3R and Hyg-Ter-F (Supplementary Table S1) were used to verify 3′-end integration. To verify that the *PpHIS3* gene had been removed from the moss genome, a PCR on the gene was performed using primers MU72his and MU73his (Supplementary Table S1). For *PpTRP1*Δ, primers MU66 TRP1-5F and 35S-R (Supplementary Table S1) were used to verify 5′-end integration of the knockout construct, and primers MU67 TRP1-3R and Hyg-Ter-F (Supplementary Table S1) were used to verify 3′-end integration. To verify that the *PpTRP1* gene had been removed from the moss genome, a PCR on the gene was performed using primers MU76trp and MU77trp (Supplementary Table S1). All PCR experiments used the ThermoFisher Scientific GeneRuler 1 kb DNA Ladder with fragment sizes of 10000, 8000, 6000, 5000, 4000, 3500, 3000, 2500, 2000, 1500, 1000, 750, 500, and 250bp.

### Construction of the Moss cDNA Expression Vector pMJ1

pMJ1 is a cDNA expression vector that was designed to either replicate episomally in moss, or integrate by homologous recombination into the non-essential moss *BS213* locus (Schaefer and Zrÿd, 1997) after digestion with *Not*I. pMJ1 was made in several steps. First, a 754 bp fragment of pCMAK1 (Hiwatashi et al., 2008) carrying the 3′ part of the *BS213* locus was amplified by PCR, adding an *Eco*RI site at the 3′ end of the fragment and a *Not*I site followed by an *Eco*RI site at the 5′ end. Similarly, a 696 bp fragment of pCMAK1 carrying the 5′ part of the *BS213* locus was amplified by PCR, adding a *Hin*dIII site followed by a *Not*I site at the 5′ end and a *Hin*dIII site at the 3′ end. The 5′ and 3′ PCR products were digested with *Hin*dIII and *Eco*RI, respectively, and cloned one after the other into the *Hin*dIII and *Eco*RI sites of the pUC119 polylinker (Vieira and Messing, 1987). The resulting plasmid, pUC119-5′-3′-BS213, is a targeting construct that will integrate anything inserted into the *Sma*I site between the 5′ and 3′ *BS213* fragments into the *BS213* locus by homologous recombination, provided that the pUC119 backbone is released by cutting with *Not*I prior to transformation into moss. pUC119-5′-3′-BS213 was linearized with *Sma*I and ligated to a 5431 bp *Pme*I-*Eco*R147I fragment of the Gateway vector pGWB2 (Nakagawa et al., 2007) containing the *nptII* kanamycin resistance gene expressed from the *NOS1* promoter and with the *NOS1* terminator, followed by a modified *CaMV35S* promoter in front of a Gateway cassette followed by the *NOS1* terminator. The resulting plasmid pMJ1 was used to make a moss cDNA expression library, which is why it has a Gateway cassette. The cassette is of no relevance in the present work since it was removed when the *PpHIS3* cDNA was cloned into pMJ1 (see below).

### Complementation of the *PpHIS3*Δ Strain by a Plasmid Expressing *PpHIS3*

The sequence of *PpHIS3* was used to search the PHYSCObase EST data base (Nishiyama et al., 2003). The cDNA clone (pdp05102) derived from *PpHIS3* was ordered from the RIKEN Bioresource Center (Ibaraki, Japan) and cloned into the TA-cloning vector pCR^®^2.1-TOPO, thus producing pTOPO-HIS3. pTOPO-HIS3 was cleaved with *Xho*I to release the *PpHIS3* insert, which was then cloned between the *Xho*I and *Eco*47III sites of pMJ1, producing pHGZ404. pHGZ404 was either left uncut or cut with *Not*I to remove the pUC119 backbone and produce a linear plasmid intended to target the integration to the non-essential *BS213* locus (Schaefer and Zrÿd, 1997). Circular and linearized plasmids were transformed into the *PpHIS3*Δ moss strain using the same method as described for the generation of *PpHIS3* knockouts, but using selection for histidine prototrophy instead of drug resistance.

### PCR Verification of Recombination and Integration of pHGZ404 into the Genome

To verified the presence of *in planta* recombination between the *NOS1* terminator PCR using the Advantage 2 PCR kit was performed on DNA from the moss transformed with pHGZ404 using primers MU83 and MU84 (Supplementary Table S1). To check if linearized pHGZ404 plasmids had integrated into the *BS213* locus through its homologous flanking sequences, a PCR was performed using primers MU BS213 5′F and MU BS213 5′R (Supplementary Table S1) to check 5′-end integration and primers MU BS213 3′F and MU BS213 3′R (Supplementary Table S1) to check 3′-end integration. To check that the *PpHIS3*Δ mutant transformed with pHGZ404 plasmid contained the *PpHIS3* cDNA expressed from the *CaMV35S* promoter that was present on the pHGZ404 plasmid, a PCR with the primers HGZ46 and HGZ58 (Supplementary Table S1) was carried out. A PCR to check if the *BS213* locus was still intact or had been disrupted was done with primers BS213 5 new2 and BS213 3 new2 (Supplementary Table S1). To check for integration into the genome by recombination between the *CaMV35S* promoter sequences present both on the plasmid and in front of the hygromycin resistance gene already present in the auxotrophic knockouts, a PCR was done using primers HGZ65-f and HGZ66-r (1334 bp) and also the primer pair MuHis3-start and MuBHRF-BS213 5 (897 bp) (Supplementary Table S1).

### Rescue of Plasmids from Moss Transformants Back into *E. coli*

Genomic DNA was extracted from moss transformants using the CTAB method (Dellaporta et al., 1983), using 1 g of chloronemal tissue grown on cellophane covered histidine-less selection plates. *E. coli* strain SF8 (*thi-1 thr-l leu-6 lacY tonA21 supE44 recBC lop-11*) was transformed with 0.02–1 μg of moss DNA and spread on to LB plates containing 150 μg/ml of ampicillin. Rescued plasmids were sequenced at Eurofins MWG Operon (Ebersberg, Germany).

### Concatemer Formation of Transformed Constructs

To check if the pBHRF-HIS3-5′-3′ knockout construct hade formed concatemers at the targeted *PpHIS3* locus, PCRs were done on genomic DNA from the *PpHIS3*Δ strains B8 and J7 using primers MU78 and MU79 (Supplementary Table S1). To check if the pBHRF-TRP1-5′-3′ knockout construct hade formed concatemers at the targeted *PpTRP1* locus, PCRs were done on genomic DNA from the *PpTRP1*Δ strains B4, N4 and N11 using primers MU107 and MU108 (Supplementary Table S1). Similarly, to check for concatemer formation in the pHGZ404 transformants, PCRs were done on genomic DNA from the transformants using primers MU83 and MU84 (Supplementary Table S1).

### Verification of the Presence of the *nptII* Marker in the pHGZ404 Transformants

To assay G418 resistance, the pHGZ404 transformants and the wild type and P5 controls were grown on 15, 20, and 30 μg/ml G418 plates for 16 days after which the results were documented. PCRs were also performed on all strains using primers NptII-F and NptII-R (Supplementary Table S1) that amplify a 786 bp fragment of the *nptII* gene.

### Plasmid Loss Experiment

To check for loss of episomally replicating DNA in the pHGZ404 moss transformants, protonemal tissue of the *PpHIS3* knockout strain transformed with either circular or linearized pHGZ404 plasmid was homogenized in a Mini Beadbeater 8 (Biospec). The homogenized material was diluted in water and plated onto BCD plates containing 5 mM ammonium tartrate (MM plates) covered with cellophane at a dilution estimated to generate approximately 25 moss colonies per plate. The plates were incubated for 2 weeks in a light chamber as described above after which the cellophane together with the moss colonies were moved to selection plates containing 50 μg/ml G418 and incubated for another week. The total number of surviving and dead moss colonies were then counted, and representative photographs were taken of each transformant.

## Results

### Construction of Histidine and Tryptophan Auxotrophic Moss Strains

**FIGURE 1:**
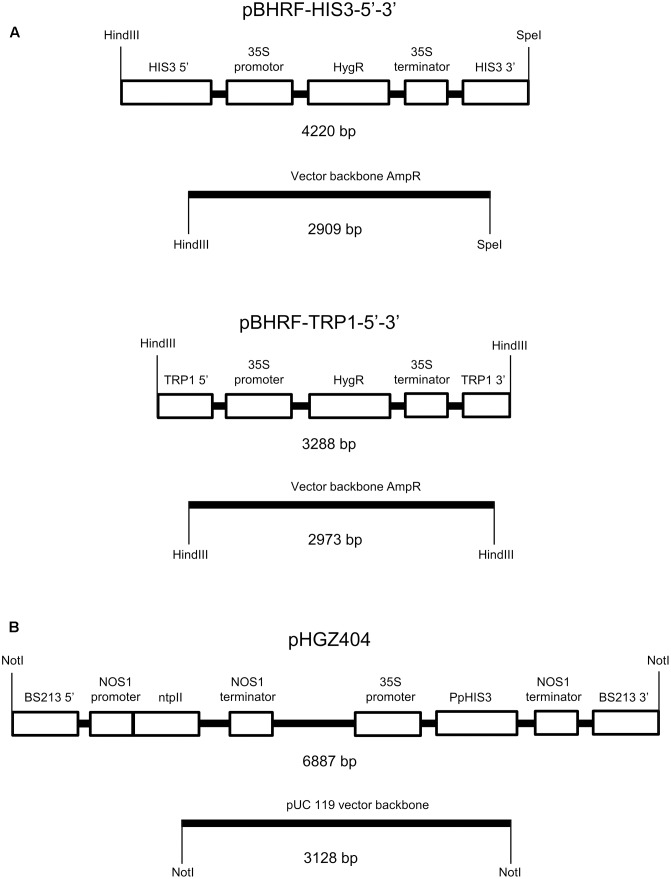
Plasmids used to make the auxotrophic *PpHIS3*Δ and *PpTRP1*Δ knockout strains and to transform the *PpHIS3*Δ strain. **(A)** The knockout plasmid pBHRF-HIS3-5′-3′ used to generate the *PpHIS3*Δ knockout strain and knockout plasmid pBHRF-TRP1-5′-3′ used to generate the *PpTRP1*Δ knockout strain. **(B)** Plasmid pHGZ404 used to transform the *PpHIS3*Δ knockout strain. Both circular (supercoiled) and linearized pHGZ404 was used to transform the moss. The linearized plasmid comprising two fragments is shown in the figure.

We searched the *Physcomitrella* genome for orthologs of several auxotrophic markers used in yeast. Many moss genes are duplicated, but we found that the moss orthologs of the yeast *TRP1* (Pp3s17_23550) and *HIS3* genes (Pp3c13_21930) are present in only one copy in the moss genome, making them suitable knockout targets. These genes will subsequently be referred to as *PpTRP1* and *PpHIS3*. The constructs used to knock out *PpHIS3* and *PpTRP1* are shown in **Figure 1A**. The constructs were designed to completely remove the open reading frames and replace them with the *hph* hygromycin resistance gene expressed from the *CaMV35S* promoter. Transformants were selected in the presence of hygromycin B on medium supplemented with the appropriate amino acid for 2 weeks, after which they were moved to non-selection plates and grown for an additional 2 weeks. The first step kills untransformed cells, and the second step allows loss of the plasmid in transient transformants. The transformants were then moved back to selection medium to identify stable transformants. Stable transformants were screened by growth on medium with and without the supplemented amino acid, to verify that they had become auxotrophic.

### Characterization of the Auxotrophic Moss Strains

**FIGURE 2:**
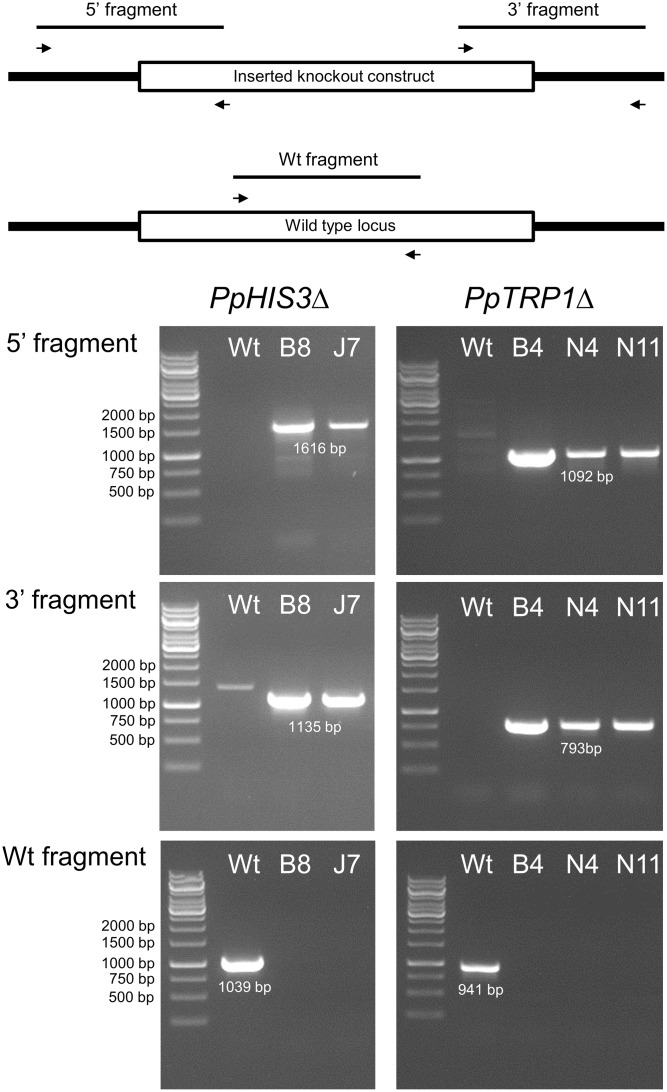
Polymerase chain reaction verification of the *PpHIS3*Δ and *PpTRP1*Δ knockouts. PCRs on genomic DNA from the *PpHIS3*Δ, *PpTRP1*Δ, and wild type (Wt) moss strains. PCRs amplifying the 5′-end region (top) and the 3′-end region (middle) are shown, in which one primer was located inside the knockout construct the other primer outside. Also shown is PCRs using two internal primers from the targeted locus (bottom) which verify that the targeted gene has been deleted in the knockout strains.

**FIGURE 3:**
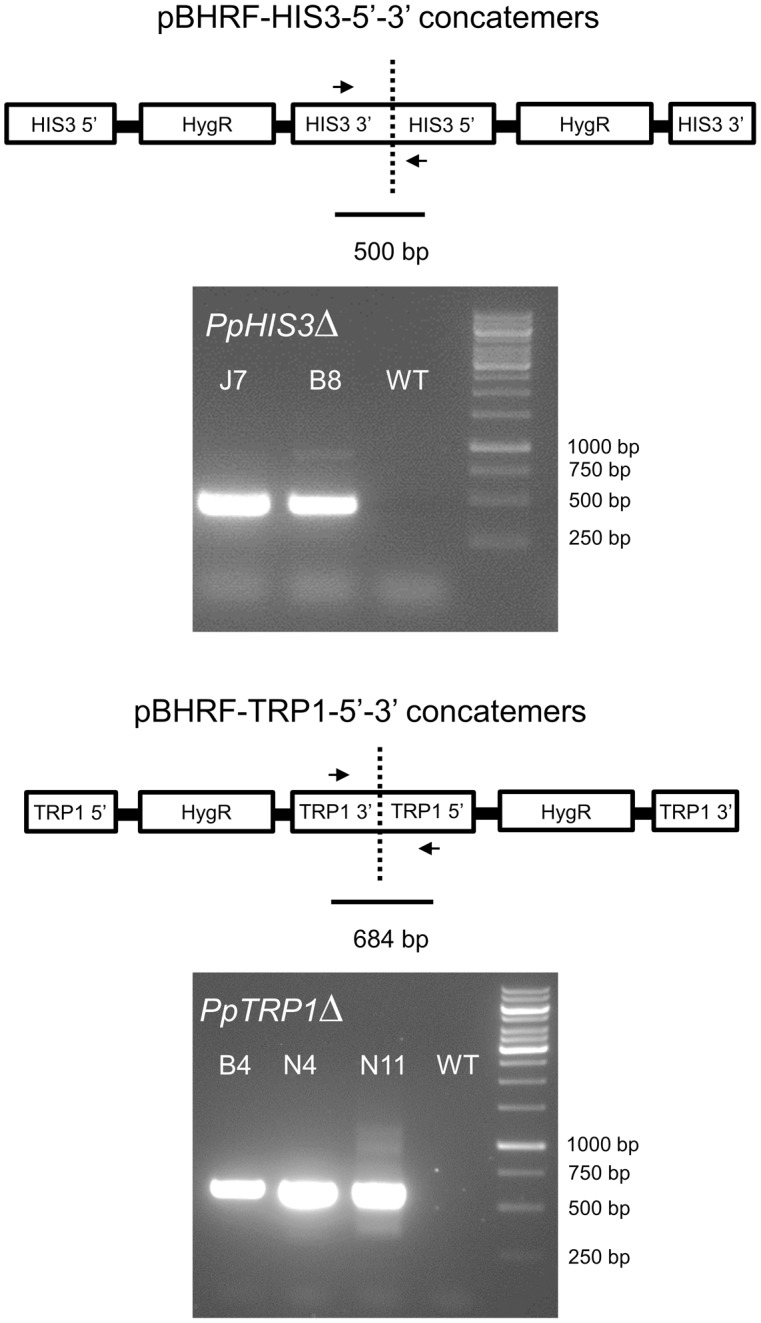
Polymerase chain reactions on genomic DNA from the *PpHIS3*Δ and *PpTRP1*Δ knockout strains to show that the knockout construct has formed concatemers. The divergent PCR primers were located in the 3′ and 5′ flanking regions of each gene that were used for targeting, in order to obtain PCR fragments that would span the concatemer junctions.

To confirm the targeted genes had been correctly knocked out we used PCR with primers located inside the *PpHIS3* and *PpTRP1* gene, respectively. Since gene targeting in moss sometimes can involve homologous recombination at one end and non-homologous recombination at the other end, PCR was also performed on the 5′-flanking and 3′-flanking region for both types of transformants with one primer located inside the knockout construct and the other primer located in the flanking region in the genome. We found four lines that were auxotrophic for histidine but only two of them (B8 and J7) had correctly integrated the *HygR* cassette by homologous recombination at both the 5′ and 3′ end of the *PpHIS3* locus (**Figure 2**). We isolated three tryptophan auxotrophs (B4, N4, and N11), all of which had the *HygR* cassette integrated by recombination at both ends of the *PpTRP1* gene (**Figure 2**). We found no auxotrophic moss colonies that did not show an alteration within the targeted locus. Gene targeting in *Physcomitrella* usually results in integration of multiple tandem copies of the knockout construct at the targeted locus (Schaefer and Zrÿd, 1997; Schaefer, 2002; Cove, 2005). Consistent with this, PCR experiments with divergent primers at each end of the knockout constructs confirmed the presence of tandem repeats of the knockout constructs in all five knockout strains (**Figure 3**).

**FIGURE 4:**
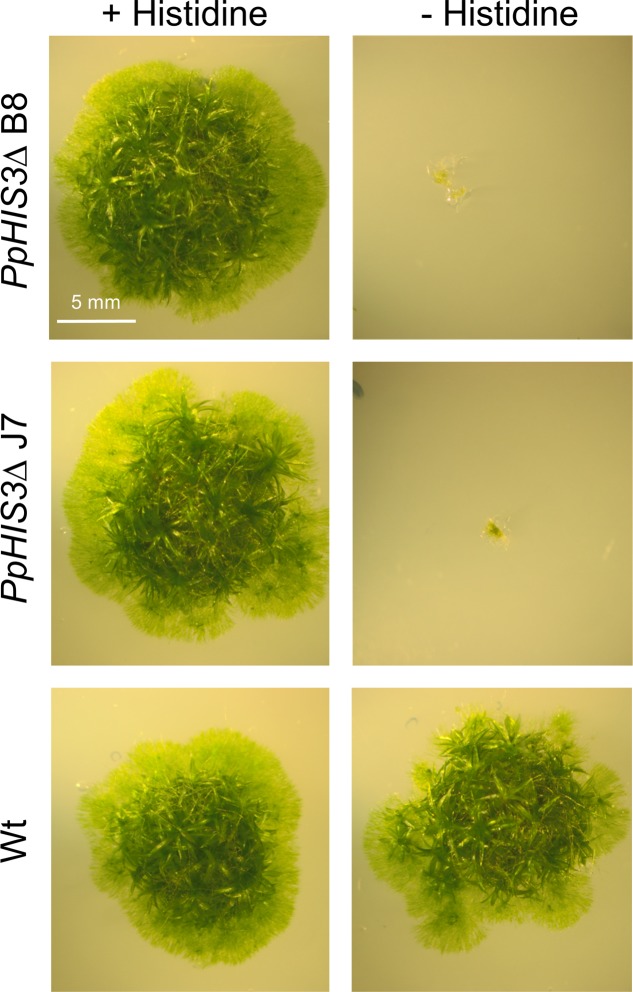
Phenotypes of *PpHIS3*Δ knockout strains. The *PpHIS3*Δ strains B8 and J7 and the Wt were grown with or without 250 μM histidine in the medium.

**FIGURE 5:**
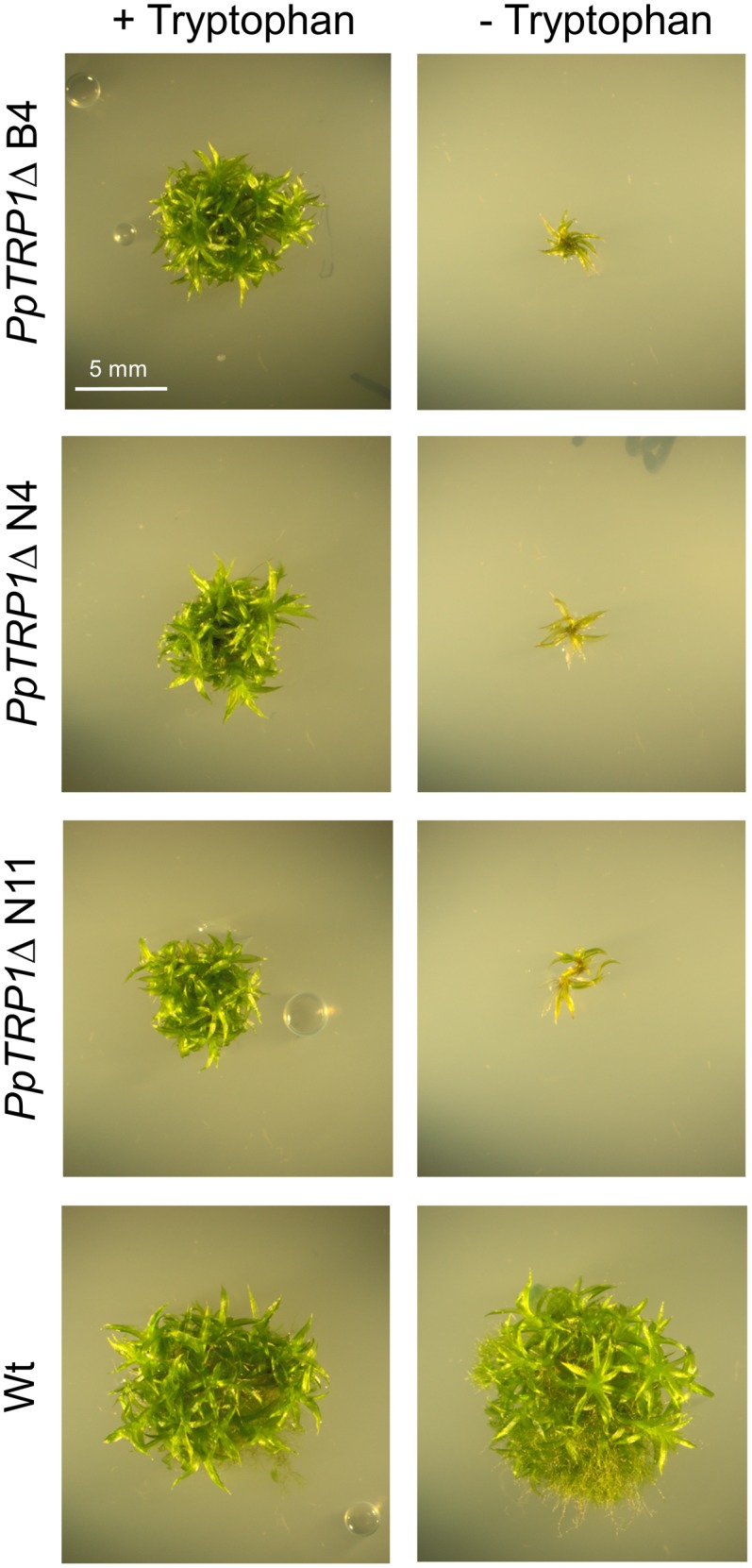
Phenotypes of *PpTRP1*Δ knockout strains. The *PpTRP1*Δ strains (B4, N4, and N11) and the Wt were grown with or without 100 μM tryptophan in the medium.

While high concentrations of histidine, and in particular tryptophan had an inhibitory effect on growth, the *PpHIS3*Δ knockouts grown on medium supplemented with 250 μM histidine had a growth rate comparable to that of the wild type. The *PpTRP1*Δ knockouts grew reasonably well when supplemented with 100 μM tryptophan even though their growth rate was much slower than that of the wild type. Without the supplemented amino acids the knockout strains were unable to grow (**Figures 4**, **5**). We further observed that the *PpTRP1*Δ strains grew best when the moss colonies were frequently moved to fresh plates. It is possible that this could due to degradation of the supplemented tryptophan, which is photosensitive and thus less stable than other amino acids. It also seemed that the *PpTRP1*Δ strains did not generate protonemal tissue to the same extent as the wild type, producing more gametophores instead. In contrast, the *PpHIS3*Δ strains grew well as long as they were supplemented with 250 μM histidine, and behaved phenotypically like the wild type. For this reason we choose to use on of the histidine auxotrophs, the *PpHIS3*Δ strain B8, in our shuttle plasmid experiments.

### Selection of Prototrophic Transformants of the *PpHIS3*Δ Moss Strain by Using the *PpHISS3* Gene on a Plasmid

We next wanted to test if the auxotrophic *PpHIS3*Δ strain could be complemented by a wild type *PpHIS3* gene and in particular if transformants could be selected by using the auxotrophic marker instead of a drug resistance marker. For this, we used the pHGZ404 plasmid containing the *PpHIS3* gene expressed from the *CaMV35S* promoter (**Figure 1B**). The plasmid also contains the G418 resistance gene *nptII* and sequences from the *Physcomitrella BS213* locus that can be used for targeted integration into the genome (Schaefer and Zrÿd, 1997). The latter requires that the plasmid is cleaved with *Not*I prior to transformation in order to release the gene targeting fragment, with the *BS213* 5′ and 3′ sequences at either end, from the pUC119 backbone (see “Materials and Methods).

In our previous study with plasmids carrying a drug resistance marker we found that circular and linearized plasmids behaved differently when transformed into moss (Murén et al., 2009). Thus, circular plasmids mostly produced unstable transformants indicating that the DNA replicated episomally, and plasmids rescued from these transformants were always identical to the original plasmid. In contrast, linearized plasmids either produced stable chromosomal integrants or highly unstable episomal transformants in which the plasmid DNA was present in a very high copy number but still lost at a high frequency (Murén et al., 2009). Moreover, plasmids rescued from moss transformed with linearized DNA differed from those rescued from circular DNA transformants in that they always contained deletions indicative of concatemer formation *in planta* (Murén et al., 2009).

In the present study, we wanted to test if these findings would hold true also for transformants obtained by selection for complementation of an auxotrophic marker. The *PpHIS3*Δ strain was therefore transformed with both circular supercoiled plasmid and plasmid linearized with *Not*I. A conceptual difference from our first study was that the linearized plasmids were expected to target the *BS213* locus instead of integrating at random points in the genome, making the molecular analysis of these events easier. Furthermore, we reasoned that stable integration into a selectively neutral locus (*BS213*) could provide an interesting alternative to episomal replication in cases where it is desirable to maintain a low copy number. As discussed below, we did see targeted integration of the linearized plasmid, but, unexpectedly, into the *PpHIS3*Δ knockout allele instead of the *BS213* locus.

**FIGURE 6:**
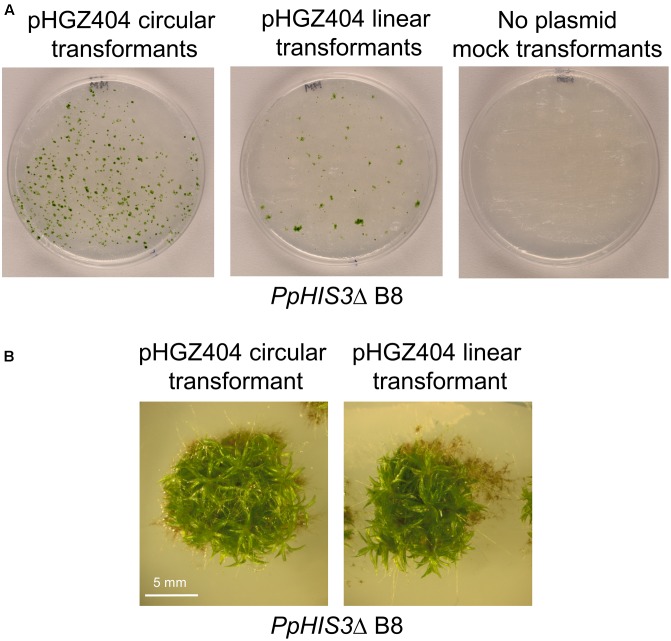
Transformation of the *PpHIS3*Δ knockout strain with circular or linearized pHGZ404 plasmid. **(A)** Agar plates with colonies obtained from the *PpHIS3*Δ strain when transformed with circular and linearized pHGZ404, as well as a mock transformation with no added DNA. Transformants were selected on histidine-less plates. **(B)** Gametophore containing colonies of the *PpHIS3*Δ knockout strain complemented with either circular or linearized pHGZ404.

The transformed cells were plated on medium lacking histidine and incubated for 3 weeks to select prototrophic transformants. As shown in **Figure 6A**, we obtained transformants with both circular supercoiled and linearized plasmid DNA. In contrast, mock transformations with no added plasmid produced no colonies. In order to eliminate transient transformants that fail to replicate the *PpHIS3* marker, individual transformants were picked to fresh histidine-less media and incubated for 2 weeks. This procedure was repeated three times. At the end, we found that 25 of 314 picked colonies from the transformation with circular plasmid DNA survived, whereas 169 of 174 picked colonies from the transformation with linearized plasmid DNA survived. This is consistent with our previous results using an antibiotic resistance marker (Murén et al., 2009), where we found that the circular plasmid initially produced more transformants than the linearized plasmid, but many of these early transformants were unstable transients that fail to replicate the plasmid DNA and eventually lose it.

**FIGURE 7:**
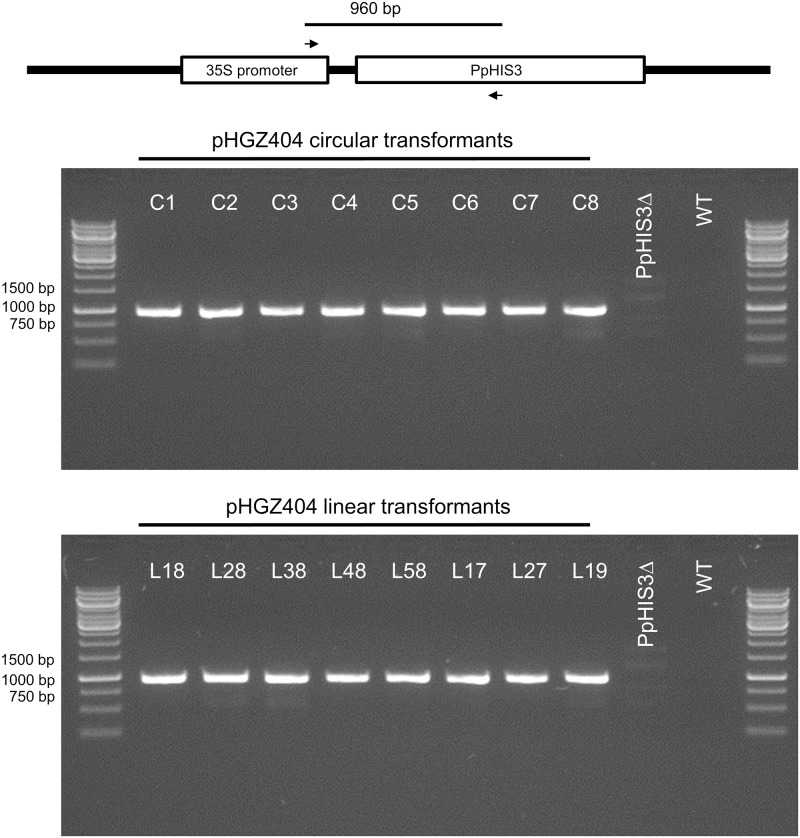
Polymerase chain reaction verification of the presence of the plasmid *PpHIS3* marker in pHGZ404 transformants. Genomic DNA from the *PpHIS3*Δ knockout strain transformed with circular or linearized pHGZ404 were checked for the presence of *PpHIS3* by amplification of a 960 bp internal fragment of *PpHIS3* expression cassette.

We picked 16 *Physcomitrella* transformants for further characterization, eight of which were obtained by transformation with circular supercoiled plasmid and eight by transformation with the linearized plasmid. To prove that the transformants had acquired the *PpHIS3* gene, we conducted PCR experiments with DNA from these transformants. All 16 transformants were positive for the presence of a fragment of the expected size (**Figure 7**). The fragment was cloned and sequenced to confirm that it was derived from the transformed *CaMV35S-PpHIS3* marker.

### Formation of Concatemers *in Planta* by Recombination between the Two *NOS1* Terminators on the Plasmid

Plasmids transformed into moss are known to frequently form concatemers, i.e., arrays of tandem repeats of transformed DNA. These arrays can comprise the entire plasmid or shorter deletion products where parts of the plasmid have been lost. We previously found that transformation with linearized DNA is particularly prone to result in the formation of large concatemers that are highly unstable, indicating that they replicate episomally (Murén et al., 2009).

We proceeded to test for the presence of concatemers using pairs of PCR primers that were designed to span across junctions in concatemers of linearized plasmid, thus amplifying a fragment that would be indicative of concatemer formation. However, we were unable to detect any such PCR products until we used internal primers located between the two *NOS1* terminators present on the pHGZ404 plasmid (**Figure 1B**). The binding site of the first primer, MU84, was placed after the *NOS1* terminator of the *nptII* gene, causing the polymerase to move upstream across the first *NOS1* terminator. The binding site of the second primer, MU83, was located within the *PpHIS3* gene, causing the polymerase to move downstream across the second *NOS1* terminator. Our rationale for testing this pair of primers was the assumption that recombination between the two *NOS1* terminators in the plasmid would occur during concatemer formation, resulting in the deletion of flanking parts of the linearized plasmid but retaining the internal fragment containing the *PpHIS3* marker that was being selected for.

**FIGURE 8:**
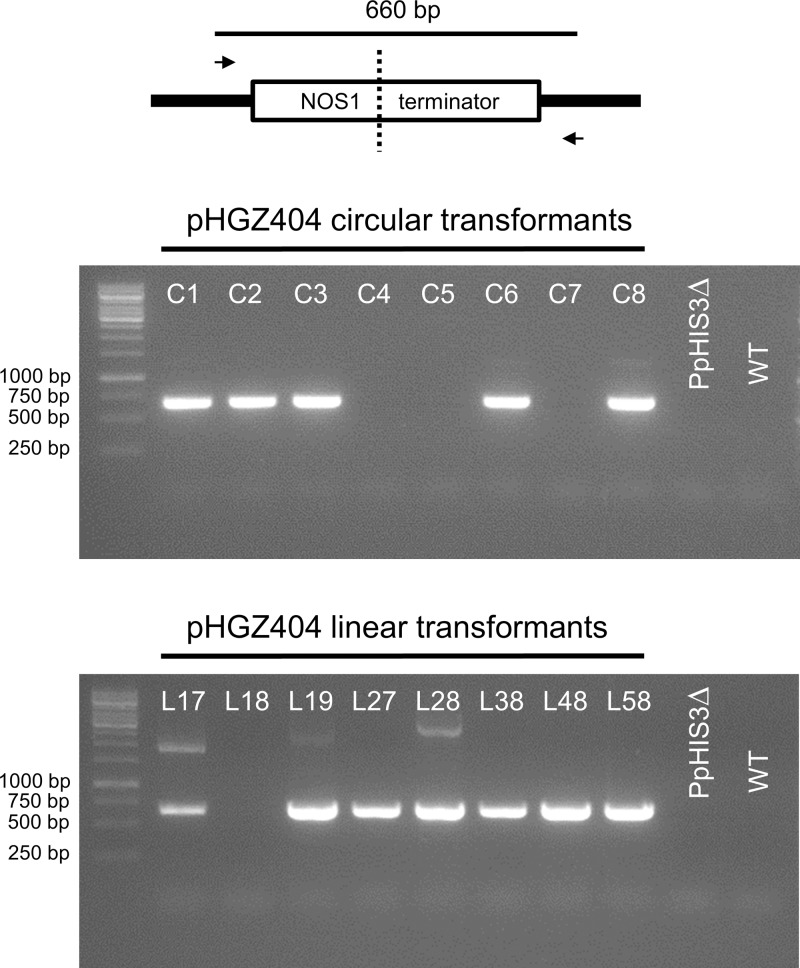
Polymerase chain reaction to confirm concatemer formation of pHGZ404 plasmids in transformants by homologous recombination between the two *NOS1* terminators. PCRs were performed on genomic DNA from the eight circular and eight linearized pHGZ404 moss transformants. The primers were located down-stream of the first *NOS1* terminator and up-stream of the second *NOS1* terminator on the pHGZ404 plasmid.

The PCR results shown in **Figure 8** confirm this assumption. Thus, among the circular DNA transformants all but C4, C5, and C7 showed bands indicative of recombination between the two *NOS1* terminators in adjacent repeats. In the linear transformants, all but L18 contained concatemer junctions created by *NOS1* terminator recombination. Recombination between the *NOS1* terminators is not the only possible mechanism for concatemer formation, and it is likely that different concatemers of pHGZ404 may exist within the transformants. That being said, the evidence of *NOS1*-mediated recombination in most of the transformants and our failure to detect junction fragments from concatemers of full-length plasmids indicates that *NOS1*-mediated recombination is a major pathway for concatemer formation in cells transformed with pHGZ404. This is consistent with our previous finding that concatemer formation in moss frequently involves recombination between repeated sequences within the transformed DNA, and not just end to end ligation of linearized plasmids (Murén et al., 2009).

### Integration of Linearized Plasmid at the Disrupted *PpHIS3* Locus

**FIGURE 9:**
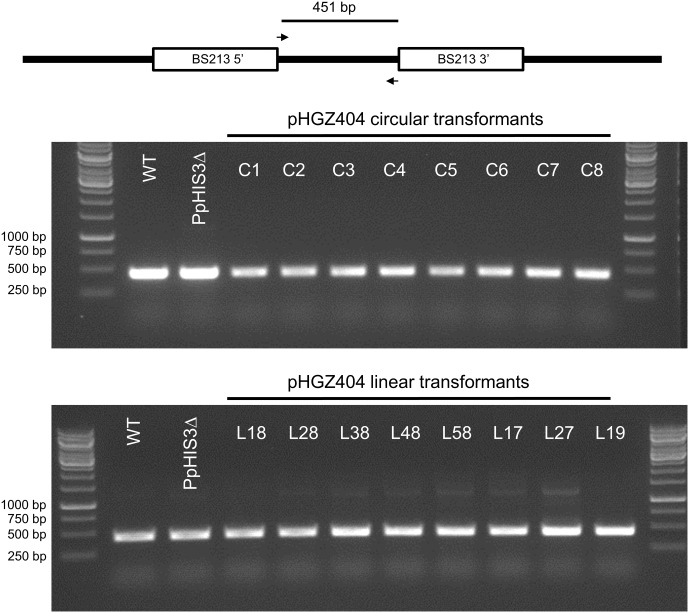
Polymerase chain reaction to check for integration at the *BS213* locus. PCRs were performed on genomic DNA from stable transformants obtained with either circular or linearized plasmids. The PCR amplifies an internal fragment of the *BS213* locus which unexpectedly was present in all transformants.

We proceeded to test if the *BS213* locus, to which the linearized plasmids would be targeted for integration, was disrupted in any of the transformants. We expected this to be true for linearized DNA transformants, but not for transformants with circular DNA. Surprisingly, we found that the *BS213* locus was intact in all 16 transformants, including those obtained with linearized DNA (**Figure 9**). Several other PCR experiments were done to check if the linearized plasmids had integrated into the *BS213* locus, but we were unable to detect any such events (data not shown). We conclude that none of the linearized plasmid transformants tested had plasmid DNA integrated into the *BS213* locus.

**FIGURE 10:**
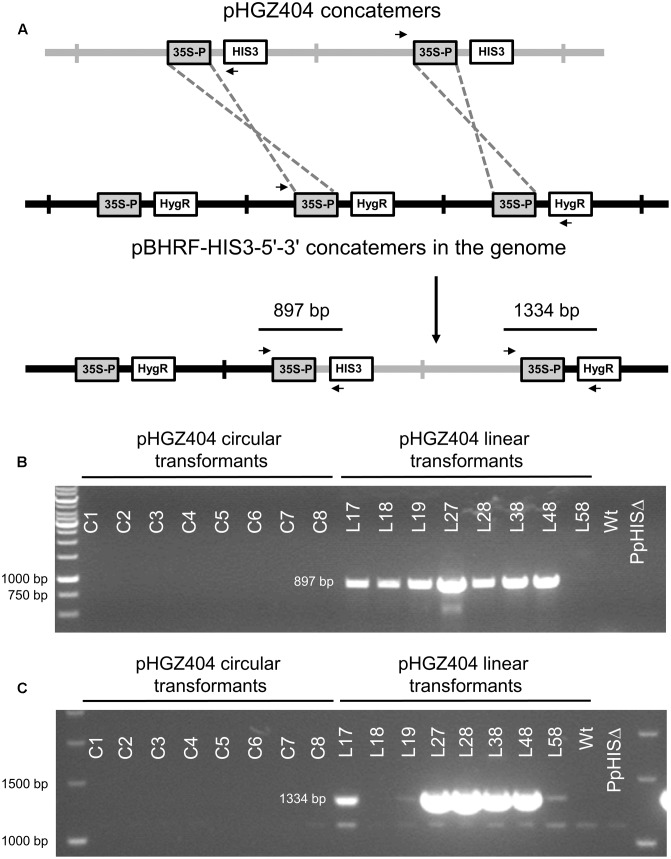
Integration of pHGZ404 concatemers into the disrupted *PpHIS3*Δ locus by homologous recombination between *CaMV35S* promoter sequences. **(A)** Proposed model for concatemer formation. A concatemer of the linearized pHGZ404 plasmid (gray) integrates into the concatemer at the disrupted *PpHIS3*Δ locus (black) by homologous recombination between *CaMV35S* promoter sequences on the plasmid and at the disrupted *PpHIS3*Δ locus. PCR primers used to check for this type of integration events are shown as *arrows*. PCRs were performed on genomic DNA from eight circular and eight linearized moss transformants with pHGZ404 in the *PpHIS3*Δ strain. 5′-end **(B)** and 3′-end **(C)** integration events were detected using one primer located within pHGZ404 and one primer located in the pBHRF-HIS3-5′-3′ knockout construct.

We therefore considered the possibility that the linearized plasmids could have integrated at some other location in the genome. Ectopic integration of transformed DNA by non-homologous recombination is less common in *Physcomitrella* (Ashton et al., 2000; Kamisugi et al., 2005, 2006), and it therefore seemed unlikely that this would have happened in all eight linear transformants tested. However, gene targeting by homologous recombination, which is a much more efficient process, could occur between the *CaMV35S* promoter in front of the *PpHIS3* gene on the plasmid and the *CaMV35S* promoter in front of the *hph* hygromycin resistance gene integrated at the disrupted *PpHIS3* locus. As previously noted, the knockout construct including the *CaMV35S* promoter is itself present as a concatemer at the *PpHIS3* locus (**Figure 3**), which would increase the likelihood of a recombination event, particularly if the incoming plasmid DNA also is a concatemer with multiple copies of the *CaMV35S* promoter (**Figure 10A**).

To test this possibility, we used pairs of primers with one primer positioned in pHGZ404 and the other in the knockout construct at the disrupted *PpHIS3* locus (**Figure 10A**). PCR with these primers showed that seven of the eight linear transformants had undergone a *CaMV35S* promoter-mediated integration event at the 5′ end of pHGZ404 (**Figure 10B**) and five of these transformants also showed evidence of a 3′ integration event (**Figure 10C**). PCR fragments from both the 5′ and 3′ integration events were cloned and sequenced to verify that integration had occurred at the disrupted *PpHIS3* locus. Only one linearized transformant, L58, showed no evidence of *CaMV35S* promoter mediated integration at the *PpHIS3* locus. In contrast, none of the eight circular transformants had any sign of integration via recombination in the *CaMV35S* promoter. We conclude that gene targeting by homologous recombination occurred with high frequency in the linearized transformants, though with a different target than intended. The possibility remains that pHGZ404 also integrated at other, unknown locations in some of the transformants, but the intended target *BS213* and the observed target *PpHIS3* are the only regions of homology to pHGZ404 in the *Physcomitrella* genome, and as noted above integration of transformed DNA by non-homologous recombination is less common in moss (Ashton et al., 2000; Kamisugi et al., 2005, 2006).

We also considered the possibility that the junction fragments detected in **Figure 10** might be formed during the PCRs by template switch recombination (Hommelsheim et al., 2014) between *NOS1* or *CaMV35S* elements on different molecules. However, this is a very unlikely explanation since these fragments were reproducibly seen in some transformants but not in others, even though all transformants contained the same plasmid DNA and the same knockout construct at the *PpHIS3*Δ locus. Thus, junction fragments consistent with integration of plasmid concatemers at the *PpHIS3*Δ locus were reproducibly seen in all linearized plasmid transformants except L58, but in none of the circular transformants on the multiple occasions that we repeated this PCR. Similarly, a junction fragment consistent with concatemer formation by recombination between the *NOS1* elements was always seen in all linearized plasmid transformants except L18, and in circular transformants C1, C2, C3, C6, and C8, but never in C4, C5, and C7. It is unlikely that this was due to failure of the PCR reactions in some samples since the PCRs in **Figures 7** and **9** did work for the same samples. These reproducible patterns, which were seen with several different primer pairs, strongly suggest that the junction fragments are present in some strains but not in others, and are not formed by template switches during the PCR.

### Episomal Replication of the Unselected *nptII* Marker

**FIGURE 11:**
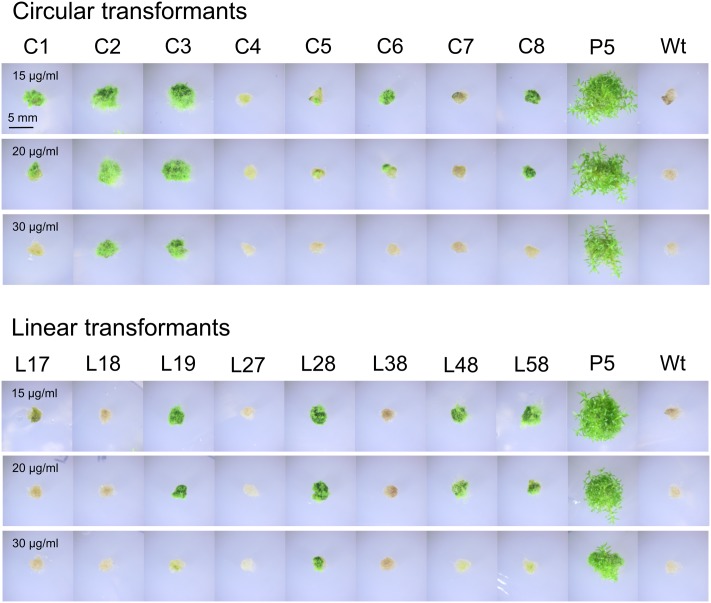
Assay for G418 resistance in different transformants of the *PpHIS3*Δ strain with plasmid pHGZ404. Eight linearized (L17–L58) and eight circular (C1–C8) pHGZ404 transformants were grown on G418-containing plates to established to what extent they retained the *nptII* drug resistance marker. Also shown as controls are the drug sensitive Wt and the stably drug resistant P5 strain, which carries chromosomally integrated copies of the *nptII* marker at the *PpHXK1* locus.

**FIGURE 12:**
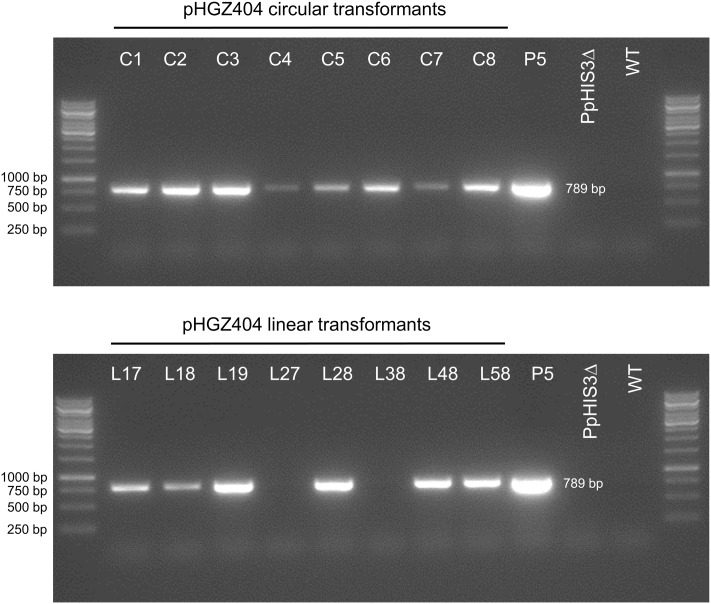
Polymerase chain reaction experiment to check for the presence of the *nptII* marker in different transformants of the *PpHIS3*Δ strain with plasmid pHGZ404. Eight linearized (L17–L58) and eight circular (C1–C8) pHGZ404 transformants were checked by PCR for the presence of the *nptII* marker. Also shown as controls are the drug sensitive Wt, the untransformed *PpHIS3*Δ strain, and the stably drug resistant P5 strain, which carries chromosomally integrated copies of the *nptII* marker at the *PpHXK1* locus.

An important question is to what extent the overall structure of the plasmid is maintained after transformation into moss, since this will affect the feasibility of plasmid rescue back into *E. coli*, for which the bacterial origin of replication and the *Amp^R^* marker must be retained. In particular, *NOS1*-mediated recombination would tend to unlink the *PpHIS3* marker, which is selected for, from the plasmid backbone and the *nptII* marker which confers resistance to G418 in moss (**Figure 1A**). To assess retention of the *nptII* marker in transformants selected for histidine prototrophy, we tested eight circular and eight linear transformants for their ability to grow on plates containing different concentrations of G418 (**Figure 11**). As a positive control, we included a knockout strain (P5) that has the *nptII* marker integrated into the *PpHXK1* gene (Olsson et al., 2003). The presence or absence of the *nptII* marker was also analyzed for each transformant by PCR of an *nptII* internal fragment, as shown in **Figure 12**.

First, we note that there was a strong correlation between resistance to G418 (**Figure 11**) and the amount of PCR product (**Figure 12**). While copy numbers cannot be accurately estimated from standard PCRs, the correlation suggests that the degree of drug resistance depends on the copy number of the *nptII* marker. In particular, we note that only two linear transformants (L27 and L38) lacked the PCR band, and they failed to grow at all three concentrations of G418. One transformant with a weak PCR band (L17) also failed to grow in the presence of G418. All other transformants showed varying degrees of resistance to G418 which correlated with the amount of PCR product. Some transformants with a weak PCR band, such as C5 and C6, showed papillation on G418, indicating that only some cells contained the *nptII* marker. This is the expected result for an episomal plasmid that is lost at high frequency (Murén et al., 2009) and suggests that at least some of the circular transformants carry such plasmids. We further note that none of the transformants grew as well as the P5 strain on G418. A possible explanation is that the latter carries multiple tandem copies of the *nptII* marker at the *PpHXK1* locus which may have been further amplified by selection on G418 plates. In contrast, our transformants were selected for histidine prototrophy and not G418 resistance.

Significantly, two linearized plasmid transformants, L27 and L38, had no trace of the *nptII* marker (**Figure 12**) even though they retained the *PpHIS3* marker (**Figure 6**). This shows that loss of unselected parts of the plasmid does occur in linearized transformants, presumably by *NOS1*-terminator mediated recombination during concatemer formation. In contrast, all eight circular plasmid transformants retained the *nptII* DNA, though in variable amounts (**Figure 12**). This is consistent with our previous finding (Murén et al., 2009) that intact plasmids can be rescued form circular but not linearized plasmid transformants (see also below).

**FIGURE 13:**
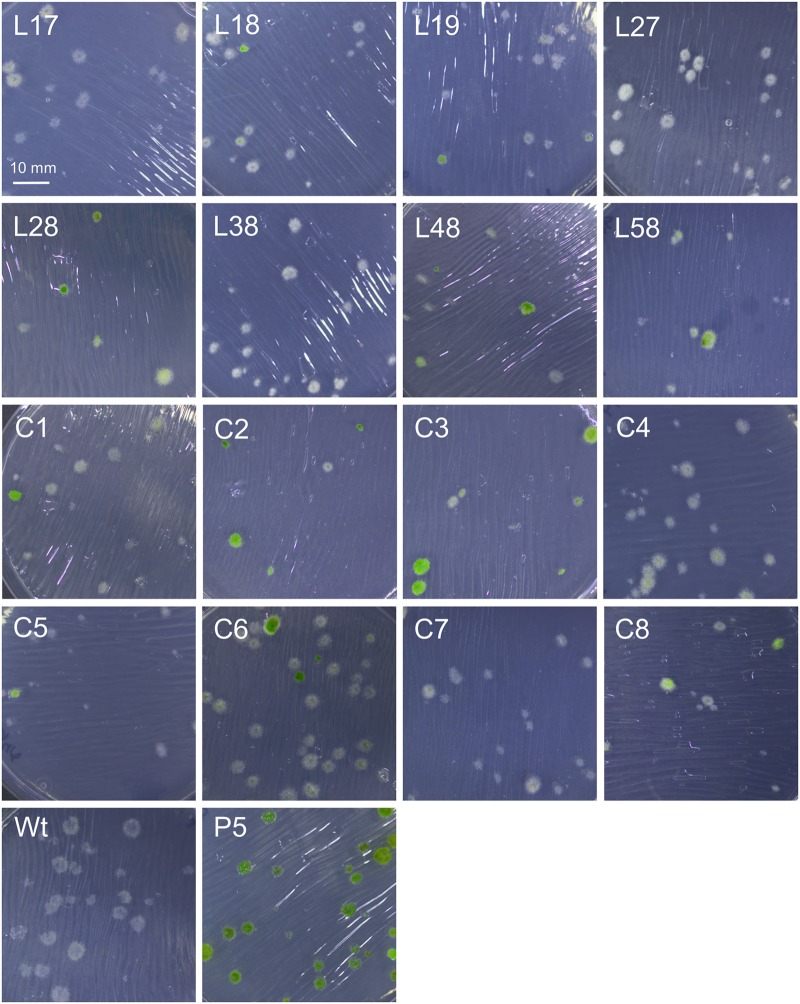
Plasmid loss experiment to check for retention of G418 resistance in different transformants of the *PpHIS3*Δ strain with plasmid pHGZ404. Representative photographs of eight linearized (L17–L58) and eight circular (C1–C8) pHGZ404 transformants after growth on G418-containing plates are shown. Colonies that survived due to retention of the episomally replicating *nptII* marker are green, and those that lost the marker and died are white. Also shown as controls are the drug sensitive Wt and the stably drug resistant P5 strain, which carries chromosomally integrated copies of the *nptII* marker at the *PpHXK1* locus.

In order to confirm that transformed DNA carrying the *nptII* marker replicated episomally, we carried out a plasmid loss experiment (Murén et al., 2009). Protonemal tissue from the different transformants was homogenized, diluted to generate an estimated 25 colonies per plate, and plated on four non-selective cellophane covered plates for each strain. After 2 weeks, the cellophane sheets were moved to G418-containing plates to select for the *nptII* marker and incubated for another week. The total numbers of surviving (green) and dead (white) colonies (**Figure 13**) were then counted for each strain.

As seen in **Table 1**, we found that all moss transformants that carried the *nptII* marker lost it at variable frequencies when grown on non-selective plates, indicating that it had not integrated into the moss genome but instead replicated episomally in these transformants. In contrast, the control *Pphxk1::nptII* knockout strain P5 (Olsson et al., 2003) retained the marker in all colonies, as expected. The frequency of marker loss varied, being lowest (7 and 19%) in transformants C3 and C2, the two transformants that grew best under selection (**Figure 11**). We conclude from these experiments that even though parts of the original plasmid including the *PpHIS3* marker integrated into the genome in some transformants, other parts including the *nptII* marker were maintained in an episomal state. In fact, none of the transformants contained a stably integrated *nptII* marker, which suggests that episomal replication is the default fate for transformed DNA in the absence of targeted integration by homologous recombination. This is consistent with the observation that episomally replicating DNA that carried a selectable marker could be maintained for several years in moss transformants without integration into the genome (Ashton et al., 2000).

**Table 1:** Plasmid loss experiment.

Moss strain	Total colonies plated	G418-resistant colonies	Loss frequency
C1	69	7	90%
C2	31	25	19%
C3	45	42	7%
C4	76	0	100%
C5	75	2	97%
C6	82	5	94%
C7	68	0	100%
C8	36	5	86%
L17	70	0	100%
L18	70	2	97%
L19	105	9	91%
L27	71	0	100%
L28	35	2	94%
L38	120	0	100%
L48	64	19	70%
L58	52	2	96%
P5	94	94	0%
Wt	50	0	No marker


*For each moss strain, the total number of plated colonies and the surviving number after transfer to G418 plates is shown. The loss frequency is the fraction of non-surviving colonies.*

### Rescue of Plasmids from Moss Transformants Back into *E. coli*

Finally, we wanted to show not only that complementation of an auxotrophic moss strain from a plasmid works, but that such plasmids can be rescued from moss transformants back into *E. coli*, thus completing the shuttle plasmid circle. We therefore extracted DNA from the transformants and transformed this DNA into *E. coli*, selecting for ampicillin resistance. The yield was lower than previously observed (Murén et al., 2009), but three plasmids could be rescued from two circular transformants, C1 and C2, and all three plasmids were identical to the original pHGZ404. This is in agreement with our previous finding that plasmid rescued from moss transformated with circular plasmids usually are identical to the original plasmid (Murén et al., 2009). It provides further support for the notion that shuttle plasmid-based methods from yeast genetics can be adapted for use in *Physcomitrella*.

**FIGURE 14:**
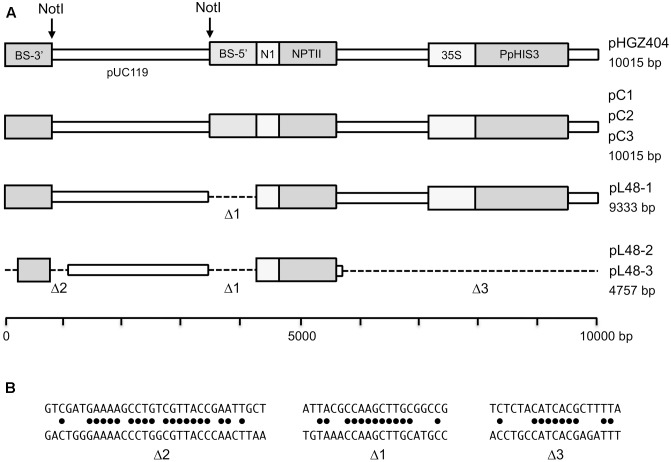
Structures of plasmids that were rescued from moss transformants back into *Escherichia coli.* **(A)** The map of the original pHGZ404 plasmid is shown at the top, and the plasmids that were rescued from moss transformants below. **(B)** The micro-homologies that were involved in the three internal deletion events.

We were also able to rescue three plasmids from one of the linearized DNA transformants, L48. This was one of the five linearized DNA transformants that had both a 5′ and a 3′ integration event at the disrupted *PpHIS3* locus, as determined by PCR (**Figure 10**). In contrast to the plasmids rescued from the circular plasmid transformants, the plasmids rescued from the linearized plasmid transformant all had deletions spanning one or both of the *Not*I sites used to cleave the plasmid prior to transformation into moss. One plasmid had a single deletion spanning the second *Not*I site. The other two plasmids, which were identical to each other, had the same deletion, but also a small deletion spanning the first *Not*I site and a big deletion that removed half of the plasmid including the *PpHIS3* marker (**Figure 14**). An examination of the sequences revealed that all three deletion events involved direct repeat recombination between short micro-homologies on the plasmid (**Figure 14**). This is consistent with previous findings when transforming moss with linearized plasmids, where deletions due to direct repeat recombination between micro-homologies frequently were observed (Kamisugi et al., 2006; Murén et al., 2009). Finally, we note that both deletions spanning a *Not*I site had the left endpoint just a few bp from the *Not*I site whereas the other endpoint was much farther away (**Figure 14**). This suggests that the deletion may have been generated by one free double-stranded end invading the other end and scanning it until a micro-homology was found. The alternative mechanism where both free ends are degraded by exonuclease until matching micro-homologies are exposed would have generated more symmetric deletions centered on the *Not*I sites.

## Discussion

We here report the construction of a histidine auxotrophic *Physcomitrella* strain in which the ortholog of the yeast *ScHIS3* gene, *PpHIS3*, was knocked out. This histidine auxotrophic strain could not survive without histidine in the growth medium, but with histidine the *PpHIS3*Δ mutant grows and behaves like wild type *Physcomitrella*. We further show that it is possible to complement this *PpHIS3*Δ knockout with a plasmid expressing a *PpHIS3* cDNA, and that such transformants can be selected on histidine-less medium, without the use of drug resistance markers. Given that it was also possible to create a tryptophan auxotroph by knocking out the *PpTRP1* gene it is reasonable to assume that other biosynthetic genes could be knocked out to create more auxotrophic markers and that these too could be complemented by plasmids expressing the relevant cDNAs. Using multiply auxotrophic strains would significantly increase the number of selection markers available in *Physcomitrella*. Today, there are only four selection markers that can be readily used in *Physcomitrella*, which confer resistance to hygromycin B, geneticine (G418), zeocin, and blasticidin S, respectively (Schaefer et al., 1991; Kasahara et al., 2004; Ishikawa et al., 2011). An additional advantage of using auxotrophic markers is that unwanted side effects of antibiotics can be avoided. It should be noted that in order to make multiply auxotrophic moss strains, it will be necessary to re-use the drug resistance markers that are used to knock out the biosynthetic genes. Furthermore, some candidate auxotrophic markers such as the *Physcomitrella* ortholog of the yeast *URA3* gene are present in two copies in the genome. Fortunately, this is now possible since the Cre-Lox system works in *Physcomitrella* (Wu et al., 2011). One could therefore make multiply auxotrophic strains with clean deletions instead of insertions of drug resistance markers at the biosynthetic loci.

### Phenotype of the *PpTRP1*Δ Knockout Mutant

Whereas the *PpHIS3*Δ mutant had no discernible phenotype except histidine auxotrophy, the *PpTRP1*Δ mutant shows both an altered phenotype and slower growth. Thus, when we sub-cultured the *PpTRP1*Δ mutant by spreading sheared moss tissue onto new agar plates it did not produce as much protonema as the wild type but instead grew slowly into small colonies containing gametophores. It is possible that the poor growth of the *PpTRP1*Δ mutant even in the presence of tryptophan is due to inefficient uptake of this amino acid from the medium. Alternatively, loss of *PpTRP1* could have an effect on the metabolism that is not remedied by the addition of tryptophan. A third possibility is that the observed phenotype, in particular the shift from protonema tissue production in favor of producing gametophores, is an effect of the added tryptophan in the plate. Tryptophan is a precursor to IAA, the most common naturally occurring variant of the plant hormone auxin (Sherwin and Purves, 1969; Zhao et al., 2002). Excess tryptophan might therefore affect the IAA level in moss. Auxin promotes the shift from chloronema to caulonema, and also stimulates the formation of buds on caulonema that eventually give rise to gametophores (Ashton et al., 1979; Jang and Dolan, 2011). The slow growth and altered phenotype of the *PpTRP1*Δ mutant would make *PpTRP1* less suitable as an auxotrophic marker, but it might still be used in cases where these effects do not matter.

### Different Fates of Circular and Linearized DNA *in Planta*

We found in our previous study using selection for a drug resistance marker that circular and linearized DNA behave differently when transformed into moss (Murén et al., 2009). Thus, circular plasmids that replicate episomally are always recovered in their original form when rescued back into *E. coli.* In contrast, linearized plasmids tend to form concatemers that either replicate episomally or integrate into the moss genome. Furthermore, linearized plasmids that are rescued back into *E. coli* always contain deletions resulting from recombination between micro-homologies within the plasmid.

The present study which used selection for an auxotrophic marker confirms and extends these findings, and provides further insights into the mechanisms of concatemer formation. Thus, we found that concatemer formation preferentially occurred by homologous recombination between the two copies of the *NOS1* terminator that are present on the plasmid (**Figure 9**). In fact, we were unable to detect concatemers resulting from simple end to end ligation of the linearized plasmid, indicating that such event are rare or non-existent *in planta*. Interestingly, we saw *NOS1*-mediated concatemer formation also in five of eight circular transformants, so this process is not limited to linearized DNA. However, it should be noted that plasmids rescued from circular transformants both in the previous (Murén et al., 2009) and the present study showed no evidence of the internal deletions associated with concatemer formation. This suggests but does not prove that intact plasmids may propagate *in planta* independently of concatemer formation.

### Unexpected Targeting of Linearized Plasmid into the *PpHIS3*Δ Locus

Surprisingly, we found that the linearized plasmid integrated into the disrupted *PpHIS3*Δ locus (**Figure 10**) instead of into the intended target, the *BS213* locus (**Figure 9**). A likely explanation for this is that the construct used to knock out the *PpHIS3* gene is present in the genome as a concatemer with multiple copies of the *CaMV35S* promoter in front of the *HygR* gene, and that the pHGZ404 plasmid also forms a concatemer prior to integration (**Figure 10A**). Accordingly, the multiple *CaMV35S* promoter fragments at the disrupted *PpHIS3* locus would present a much larger and therefore more likely target for homologous recombination than the single copy of the *BS213* target locus. This finding is consistent with our previous observations suggesting that when different sequence homologies are available, homologous recombination in *Physcomitrella* almost always proceeds by using the most extensive sequence homology. Thus, we found that when a plasmid carries two copies of a 17 bp sequence, deletions within the linearized plasmid always occurs by recombination between these two sequences, even though shorter micro-homologies also are available (Murén et al., 2009). Similarly, as discussed above, concatemer formation in the present study occurs largely or exclusively by recombination between the two *NOS1* terminators on the plasmid. It should be noted that this was true even for those circular plasmid transformants in which concatemer formation could be detected. We conclude from these findings that it is important to design shuttle plasmids and targeting constructs for use in moss in such a way that undesired recombination events are minimized. In particular, repeated sequences on plasmids should be avoided, and a different promoter such as the *NOS1* promoter should be used for expression of the auxotrophic marker. Alternatively, the above discussed use of the Cre-Lox system to generate multiply auxotrophic moss strains would prevent undesired targeting since it would leave no *CaMV35S* promoter sequences in the genome.

### Plasmid Rescue from Circular and Linearized Plasmid Transformants

All three plasmids rescued from circular plasmid transformants were identical to the original pHGZ404 plasmid. This confirms our previous finding that the original plasmid can be rescued from moss transformed with a circular plasmid (Murén et al., 2009), and suggests that this is a general result that is not dependent on the marker used for selection, which will facilitate further work with shuttle plasmids in moss. In contrast, the three plasmids rescued from one of the linearized plasmid transformants all had internal deletions, which had been generated by recombination between micro-homologies within the plasmid (**Figure 14**). This is also consistent with our previous finding that linearized plasmids are re-ligated *in planta* by direct repeat recombination between short micro-homologies on the plasmid (Murén et al., 2009).

The frequency of plasmid rescue was lower than previously observed (Murén et al., 2009) for both types of transformants. A possible reason is the presence of the two *NOS1* terminators in pHGZ404, which recombine during concatemer formation *in planta*. Such recombination will unlink the *PpHIS3* marker, which is being selected for, from the plasmid backbone that carries the bacterial origin of replication and the ampicillin resistance gene. Consistent with this, we found that the *nptII* marker was present in variable amounts, and was completely lost in some transformants (**Figure 12**). We note that none of the plasmids rescued from the linearized plasmid transformants consisted of just the re-ligated plasmid backbone. This suggests that end-to-end ligation is rare *in planta*, and that recombination and concatemer formation is the major pathway for processing of linearized DNA in moss. However, we rescued only three plasmids from the linear transformants, and more plasmids need to be examined before this type of event can be ruled out.

It should be emphasized that our ability to rescue circular plasmids identical to the original plasmid back into *E. coli* does not necessarily mean that the plasmid replicated as a single copy circular plasmid *in planta*. As previously discussed (Murén et al., 2009) it is conceivable that the transformed DNA replicates as a concatemer from which single copy plasmids pop out by homologous recombination. What is important is not how the transformed plasmid replicates but that it can be rescued back into *E. coli* without rearrangements or deletions since this makes it possible to use molecular genetics methods based on shuttle plasmids in moss.

### Use of Shuttle Plasmids in Moss Molecular Genetics

The fact that we could complement the *PpHIS3*Δ knockout mutant with *PpHIS3* expressed from a plasmid implies that cloning of genes and cDNAs by complementation from plasmid libraries is possible in *Physcomitrella*. This is very interesting since it would not just enable cloning of novel genes by complementation, but may also make dosage suppressor screens with plasmid libraries possible, just as in yeast. By transforming a *Physcomitrella* mutant with a cDNA library and screening for plasmids capable of suppressing the phenotype of the mutant, one might thus isolate functionally related genes. This is a powerful molecular genetic tool that is used extensively in yeast genetics (Rine, 1991), but its use in other organisms has been hampered by the lack of suitable shuttle plasmids. Dosage suppressor screens in *Physcomitrella* would make it possible to study plant specific genes and functions, such as photosynthesis, that are impossible to study in yeast.

There are also other methods from yeast molecular genetics that could be adapted for use in moss. One such method is plasmid shuffling, in which a plasmid with an essential wild type gene is replaced by a plasmid carrying a conditional mutant allele of the same gene. Plasmid shuffling enables easy selection of new temperature-sensitive mutations and other conditional mutations in essential genes. This method relies on the ability to select against a marker on the first plasmid, so that its loss is forced. The most commonly used negative selection marker in yeast is the *URA3* gene, which can be selected both for, on uracil-less medium, and against, on 5-fluoroorotic acid containing medium. In the *Physcomitrella* genome there are two *URA3* orthologs. It would therefore be interesting to make a double knockout of the two moss *URA3* orthologs, and test if a *PpURA3* gene on a plasmid can be used for both positive and negative selection. Other possible auxotrophic markers are the orthologs of the yeast *ADE2* and *LEU2* genes, both of which exist in single copies in *Physcomitrella*.

## Conclusion

Adapting methods and tools from yeast to the model plant *Physcomitrella* holds great promise. With the successful complementation of the *PpHIS3*Δ knockout strain we are one step closer to a working moss shuttle vector system. This will facilitate the adaption of additional tools and methods from yeast to moss and thus expand the boundaries for what is currently possible in plant research. Finally, we note that these methods also could be used in other plants where homologous recombination is efficient, such as the moss *Ceratodon purpureus* (Brücker et al., 2005; Trouiller et al., 2009).

## Author Contributions

MU and HR conceived the study, designed the experiments, and wrote the manuscript. MU, G-ZH, and MJ did the experimental work. All four authors read and approved the manuscript.

## Conflict of Interest Statement

The authors declare that the research was conducted in the absence of any commercial or financial relationships that could be construed as a potential conflict of interest.

## Abbreviations

IAA: indole-3-acetic acid
PCR: polymerase chain reaction
Pp: *Physcomitrella patens*
Sc: *Saccharomyces cerevisiae*
