# Supplementary material for: Testing of Auxotrophic Selection Markers for Use in the Moss Physcomitrella Provides New Insights into the Mechanisms of Targeted Recombination
Source: Front Plant Sci. 2017 Nov 3;8:1850. doi: 10.3389/fpls.2017.01850 (PMC5675891; doi:10.3389/fpls.2017.01850)
Supplement: Supplementary file 1 [file Table_S1.PDF]

Table S1. Oligonucleotides used.

Name	Sequence
MU64	5'-CAATCAGAAGTAACAGAGTTGTGTAACATT-3'
MU65	5'-CTCGAGCTCCAGAGCAAGCCAG-3'
MU63	5'-GCGGCCGCAGTAAAGTTCTTGATTTTT-3'
MU57 HIS3-3R	5'-AGATCTATCACGTTCCTATTTCAGTATTTG-3'
MU59	5'-AAGCTTGAGCTGAGATTCTCAAAAGG-3'
MU47 TRP1-5R	5′-CTCGAGCTTCCAAACCCCCGAACAA-3'
MU60	5′-GCGGCCGCTGGTGATAGATTCA-3′
MU49 TRP1-3R	5′-AGATCTCATTTTGAAGAGAACATGAAGCTT-3′
MU70 HIS3-5F	5′-CAGCGCTCATAATTGTTCTACAAC-3′
MU71 HIS3-3R	5′-ATGTGGCTCTGGCAACTTTGAA-3′
MU66 TRP1-5F	5′-GGTGATGGAGGACATAGAAGC-3'
MU67 TRP1-3R	5′-CCTTGAGTAACACAAATGTGTATTTG-3'
35S-R	5′-AGGTCCTCTATTTGAATCTTTGACT-3'
Hyg-Ter-F	5′-TGAAATCACCAGTCTCTCTCTAC-3'
MU72his	5′-TTATCTGTGGTCGTAATTAGAGATCTT-3'
MU73his	5′-AAGCTGCATCACAGAGTGATTCTTA-3'
MU76trp	5′-GTATTCGAATTTTAGAATTTGATGAGAG-3'
MU77trp	5′- CATTGAACAAGTCAGAATTCTGCAC-3'
MU BS213 5'F	5′-AGGTTCGGTCAAGGATGGTTAC-3'
MU BS213 5'R	5′-CAATAATGGTTTCTGACGTATGTGC-3'
MU BS213 3'F	5′-CGGTGTCATCTATGTTACTAGATC-3'
MU BS213 3'R	5′-GCACATTGCAGCGGTACTTTAGA-3'
HGZ46	5′-ACGTTCCAACCACGTCTT-3'
HGZ58	5′-CAACACTCGTCTCTTTGG-3'
BS213 5 new2	5′-TGTAAACCCAAACTTGGTGTCG-3'
BS213 3 new2	5′-CGCTACTTCTAAGTTTAGGATTC-3'
MuHis3-start	5′-ATTGTGGACAAGTCTACAACAACAA-3'
MuBHRF-BS213 5	5′-TCAGGCGTCAAACACGAGTATAA-3'
MU78	5′-ACTGATGATAAAGTATCTTAAGTATTGATA-3'
MU79	5′-AACACAAATAAGTTCAGCGAGCC-3'
MU83	5′-ATCAAATGGAATTAGATGTCACACCATCA-3'
MU84	5′-GCCGCCAGCATTGACAGGAG-3'
NptII-F	5′-TTGAACAAGATGGATTGCACGCAG-3'
NptII-R	5′-AGAACTCGTCAAGAAGGCGATAGAA-3'
HGZ65-f	5′-TCATTGGTGACGTTTCCG-3'
HGZ66-r	5′-AACTTCTCGACAGACGTC-3'
MU107	5′-TCGTAACATCAAGTTTCGTACTCTCT-3'
MU108	5′-TTTGCCTATCAGCTACTCCAGTG-3'
